# A preliminary study of the immunogenic response of plant-derived multi-epitopic peptide vaccine candidate of *Mycoplasma gallisepticum* in chickens

**DOI:** 10.3389/fpls.2023.1298880

**Published:** 2024-01-23

**Authors:** Susithra Priyadarhni Mugunthan, Divyadharshini Venkatesan, Chandramohan Govindasamy, Dhivya Selvaraj, Harish Mani Chandra

**Affiliations:** ^1^ Department of Biotechnology, Thiruvalluvar University, Vellore, Tamil Nadu, India; ^2^ Department of Community Health Sciences, College of Applied Medical Sciences, King Saud University, Riyadh, Saudi Arabia; ^3^ Artificial Intelligence Laboratory, School of Computer Information and Communication Engineering, Kunsan National University, Gunsan, Republic of Korea

**Keywords:** plant made vaccines, *Mycoplasma gallisepticum*, multi-epitope, Avian mycoplasma, plant expression system

## Abstract

*Mycoplasma gallisepticum* (MG) is responsible for chronic respiratory disease in avian species, characterized by symptoms like respiratory rales and coughing. Existing vaccines for MG have limited efficacy and require multiple doses. Certain MG cytoadherence proteins (GapA, CrmA, PlpA, and Hlp3) play a crucial role in the pathogen’s respiratory tract colonization and infection. Plant-based proteins and therapeutics have gained attention due to their safety and efficiency. In this study, we designed a 21.4-kDa multi-epitope peptide vaccine (MEPV) using immunogenic segments from cytoadherence proteins. The MEPV’s effectiveness was verified through computational simulations. We then cloned the MEPV, introduced it into the plant expression vector pSiM24-eGFP, and expressed it in *Nicotiana benthamiana* leaves. The plant-produced MEPV proved to be immunogenic when administered intramuscularly to chickens. It significantly boosted the production of immunoglobulin Y (IgY)-neutralizing antibodies against cytoadherence protein epitopes in immunized chickens compared to that in the control group. This preliminary investigation demonstrates that the plant-derived MEPV is effective in triggering an immune response in chickens. To establish an efficient poultry health management system and ensure the sustainability of the poultry industry, further research is needed to develop avian vaccines using plant biotechnology.

## 1 Introduction

Vaccination against *Mycoplasma gallisepticum* was first recommended by Adler et al. (1960) and used as a control measure for mycoplasmosis in poultry. Currently available vaccines for the control of chronic respiratory disease (CRD) in chickens are either killed whole cells (bacterins) or live attenuated strains. Furthermore, the currently available live attenuated vaccination for this CRD is ineffective in controlling the disease, and there is a risk of virulence re-emergence in *M. gallisepticum*-infected chickens (Ferguson-Noel et al., 2012; Ishfaq et al., 2020). With an increased antimicrobial resistance and abridged antibiotic efficacy to control *M. gallisepticum* (MG) infections, control of CRD is often coupled with novel and effective vaccines and improvement of existing vaccines.

Commercially bacterins/inactivated and live attenuated vaccines have been used in the prevention of *M. gallisepticum* infection. The R strain has reportedly been accessible for many years and has been shown to reduce *M. gallisepticum*-related production loss, egg transmission, and respiratory tract lesions (Ferguson-Noel et al., 2012). Various strains like strain F (CEVAC MG F), strain K (VAXXON® MG Live), strain ts-11 (VAXSAFE MG), strain 6/85, and strain S6 (MG-Bac vaccine) are commercially available globally (Mugunthan et al., 2023). The challenges in current *M. gallisepticum* vaccines are that live vaccines frequently demonstrate pathogenicity and adverse effects, but bacterins are expensive and frequently require repeated doses to boost avian immune systems (Ishfaq et al., 2020).

The majority of contemporary vaccine development tactics are based on single or multiple antigens. A sequence of epitopic (antigenic) peptides make up a multi-epitopic vaccine, which helps prevent infection by eliciting an immune response. A multi-epitopic vaccine should have epitopes that can stimulate the immune system for the production of cytotoxic and helper T lymphocytes as well as B cells against the intended pathogen (Zhang, 2018). Vaccinations based on multi-epitopes have advantages over traditional vaccinations. They take less time to develop and do not involve microbial cultivation. In contrast to live attenuated strains, epitope-based vaccinations reduce the possibility of virulence reversal. Due to their small size, the epitopes can also be perceptively altered and optimized to maximize their efficiency in eliciting stronger immune responses and have increased stability. Since they are highly specialized and stable and do not involve complete microbes, they provide safety. The vaccine candidate can bind numerous human leukocyte antigen (HLA) alleles simultaneously due to the existence of multiple epitopes, ensuring the appropriate immunological response in a diverse population. Multi-epitopic vaccines have been developed for the following poultry disease pathogens: Newcastle disease virus, avian influenza A (H7N9) virus, and *Eimeria* parasite (Sathish et al., 2017; Hasan et al., 2019; Venkatas and Adeleke, 2019; Madlala et al., 2021; Mugunthan and Harish, 2021; Mugunthan and Mani Chandra, 2021). Due to their specificity, epitope-based vaccines are gaining popularity. Although more research and validation are needed, the utilization of recombinant vaccine technology can provide a secure and effective vaccination against MG infections.

Currently, bacterial, yeast, and mammalian expression systems represent the vast majority of the vaccine expression systems used in the development of multi-epitopic vaccines. These traditional vaccine production methods have a number of limitations. For instance, challenges expressing higher eukaryotic proteins, endotoxin buildup, and host contamination with proteases were issues with the bacterial expression method. For the past two decades, apart from the conventional production platforms and technologies used in manufacturing vaccines, drugs, and other biologics by industries and pharmaceutical companies, plant-based production systems have gained attention for vaccine development due to exhilarating prospects and possibility of developing edible vaccines for veterinary diseases, which have the potential to produce safe, effective, stable, and economical prophylactics, vaccines, and medicines for a variety of ailments, including infectious disorders, in large-scale production at a low cost and with no chance of contamination. The most efficient plant host for the production of recombinant proteins is *Nicotiana* spp., which also yields a large amount of biomass in addition to the highest transient concentrations of recombinant proteins (Gray, 1978; Bano et al., 2021; Gómez, et al., 2013). Plant-produced vaccines prioritize demonstrating proof of concept and effectiveness. The recent progress in creating resilient, stable, and temporary plant production systems for vaccine antigens holds implications for veterinary medicine. Moreover, there is the potential for plant-produced vaccines to contribute to the advancement of both animal and potentially human health within the framework of the One Health perspective.

Plant-based vaccines offer several potential benefits, including the ability to achieve high production yields, rapid manufacturing, safety, and cost-effectiveness. Additionally, these vaccines are less likely to be contaminated with uncommon mammalian pathogens (Peyret et al., 2021). Consequently, the idea of using plant-based platforms as an alternative solution to challenges associated with animal-based methods has been under consideration for a while. It is feasible to express viral proteins like the SARS-CoV-2 spike protein within plants, leading to the formation of viruslike particles (Jung et al., 2022). A significant development in this area is Medicago Inc.’s accomplishment of phase 3 clinical trials for a flu vaccine derived from *Nicotiana benthamiana*. This plant-based quadrivalent viruslike particle (QVLP) flu vaccine exhibited efficacy comparable to that of existing vaccines (Ward et al., 2020; Ward et al., 2021). Notably, Medicago’s plant-based COVID-19 viruslike particle vaccine (Covifenz®) was granted approval by Health Canada in February 2022, marking it as the first approved plant-based vaccine (Hager et al., 2022). Recently, Lucero et al. (2021) documented that an oral plant-based vaccine for infectious bursal disease elicited a protective immune response in chickens. The utilization of plants as a novel platform for producing protein-based drugs has garnered considerable attention. Epitope-based vaccines offer several advantages, such as safety, precision, and scalability, but they also face challenges related to the design of effective epitopes, limited immune responses, and potential difficulties in achieving herd immunity.

Hence, in this study we cloned the *M. gallisepticum* multi-epitopic vaccine (MEPV) gene in the pSiM24-eGFP plant expression vector, and by *Agrobacterium*-mediated transient expression, the MEPV protein is expressed in plants. To our knowledge, this is the first study to express an MEPV from *M. gallisepticum* in plants. The protein expression is validated by sodium dodecyl sulfate–polyacrylamide gel electrophoresis (SDS-PAGE) and Western blotting. Furthermore, ELISA confirmed the immunogenic nature of plant-expressed MEPV protein by eliciting antibody response against MEPV.

## 2 Materials and methods

### 2.1 Construction of the MEPV plant expression vector

**Figure 1:**
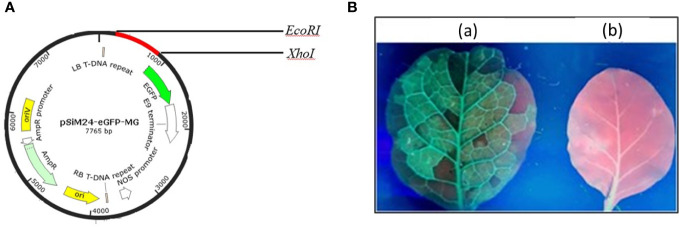
**(A)** Vector map showing the MEPV gene construct (red color) in the pSiM24-eGFP vector. **(B)** Green fluorescent protein (GFP) expression in an *N. benthamiana* leaf seen under a UV illuminator: **(A)** GFP-expressing leaf; **(B)** control.

The genetic components of the binary vector pSiM24 are as follows: left and right T-DNA borders, a full-length transcript promoter (M24) of the Mirabilis mosaic virus with duplicated enhancer domains, three multiple cloning sites (Sambrook et al., 1989), a 39rbcsE9 terminator, replication functions for *Escherichia coli* (ColE1) and *Agrobacterium tumefaciens* (pRK2-OriV), and the replicase (bla) (Dey and Maiti, 1999; Haq et al., 1995; Hofgen and Willmitzer, 1988; Sahoo et al., 2013). The MEPV candidate from the epitopic regions of GapA, CrmA, Hlp3, and PlpA was designed (Mugunthan and Harish, 2021). The nucleotide sequence of MEPV was codon optimized and commercially synthesized (Synbio Technologies LLC) with *EcoRI*, *AgeI*, and *XhoI* restriction sites for cloning at the 5′ and 3′ ends, respectively. The MEPV region was ligated into the pSiM24-eGFP vector (Sahoo et al., 2014) using *EcoRI* and *XhoI* restriction sites to construct the plant expression vector pSiM24-eGFP-MEPV (**Figure 1A**). The ER retention signal peptide (KDEL) was included at the C-terminus of the gene construct.

### 2.2 Expression of the MEPV candidate in *N. benthamiana* by agroinfiltration

The plant expression vector pSiM24-eGFP-MEPV was transformed into *A. tumefaciens* strain GV3101 cells using the freeze-thaw method (D’Aoust et al., 2008). The recombinant *A. tumefaciens* clones were confirmed by polymerase chain reaction (PCR) using MEPV gene-specific primers. *A. tumefaciens* containing pSiM24-eGFP-MEPV was resuspended with 1× infiltration buffer (10 mM 2-(*N*-morpholino)-etanesulfonic acid (MES), 10 mM MgSO_4_, at pH 5.5) to get a final OD_600_ of 0.2 prior to agroinfiltration. The *A. tumefaciens* suspension was injected into the adaxial side of 36-day-old *N. benthamiana* leaves. The infiltrated plants were maintained in an optimal 16-h light/8-h dark condition at 28°C and harvested after 1 day to 6 days post infiltration (dpi).

### 2.3 Extraction and purification of plant-produced MEPV protein

Infiltrated plant and control leaf samples (negative control) (1 g) were homogenized with 500 µl of the extraction buffer [phosphate-buffered saline (PBS) (NaCl 8.00 g, KCl 0.20 g, Na_2_HPO_4_ 1.44 g, KH_2_PO_4_ 0.24 g, H_2_O 800 ml, adjust pH to 7.4 with HCl or NaOH) + 0.1% Triton X-100] using a mortar and pestle and stored in ice. The protein content was quantified according to Lowry et al. (1951).

Protein purification (column based) was achieved using the presence of N-terminal His tag in the recombinant MEPV protein. The protein from the highest expression day was used for purification. The purified protein was isolated and quantified according to Lowry et al. (1951).

The purified plant-produced pSiM24-eGFP-MEPV protein was analyzed by using SDS-PAGE and Western blotting. For Western blot analysis, the proteins were separated in SDS-PAGE gel and the gel was equilibrated in transfer buffer and transferred to a nitrocellulose membrane (Pall Life Sciences BioTrace™) using the semidry transfer technique at 0.8 mA/cm^2^ and 45 V, for 90 min. Furthermore, the protein was analyzed using a primary antibody (anti-polyhistidine antibody) and a secondary antibody [anti-mouse immunoglobulin G (IgG) antibody conjugated with horseradish peroxidase (HRP)].

### 2.4 Chicken immunization

The purified plant-produced MEPV proteins at doses of 5 μg, 10 μg, 15 μg, 20 μg, and 25 μg protein were formulated with Freund’s complete adjuvant (G-Biosciences) to a total volume of 0.5 ml and injected into a specific pathogen-free (SPF) 21-day-old chicken’s pectoral muscle (Evans et al., 2009). PBS containing Freund’s adjuvant without the antigen was used as a control. A total volume of 0.5 ml of the vaccine was used for immunization. The blood samples were taken via puncture of the *vena ulnaris* and collected in 2-ml tubes on days 7, 14, 21, 28, 35, 42, and 49 post immunizations. The samples were cooled, and the serum was separated and stored at −20°C until further use.

### 2.5 Evaluation of antibody response by ELISA

The collected chicken serum was used to quantify the presence of anti-MEPV-specific immunoglobulin Y (IgY) antibodies using ELISA. The purified MEPV protein was diluted in 0.05 M carbonate buffer (pH 9.6) to a final concentration of 0.5 µg/ml, and 100 µl from this dilution was added to each well of an ELISA plate and incubated at 4°C overnight for coating. Next, 100 µl of serum from immunized and control chickens diluted 1:400 in blocking buffer was added to each well and incubated for 30 min at 37°C. The wells were washed, and 100 µl of HRP-conjugated anti-chicken IgY antibodies (Abcam) at a 1:10,000 dilution in PBS was added and incubated at 37°C for 30 min. After incubation, the wells were washed thrice and 100 µl of 3,3′,5,5′-tetramethylbenzidine (TMB) substrate was added and incubated for 15 min. Once the color was developed, 100 µl of 2 M H_2_SO_4_ was added and the optical density at 450 nm was determined by an ELISA reader (Bio-Rad). All experiments were performed in duplicate, and 1× PBS was used as a control. The antibody titers were compared by GraphPad Prism 9 using Tukey’s multiple-comparison test.

### 2.6 Statistical analysis

GraphPad Prism 9 (GraphPad Software, Inc.) was employed to perform a two-way analysis of variance (ANOVA) and multiple comparisons to determine the statistical significance across groups. Geometric mean titer (GMT) and a 95% confidence interval (CI) were used to depict the results.

## 3 Results

### 3.1 Transient expression of MEPV protein in *N. benthamiana*

The codon-optimized MEPV gene was cloned into plant expression vector pSiM24-eGFP. For expression of MEPV protein in plants, *N. benthamiana* plants were infiltrated with *A. tumefaciens* containing pSiM24-eGFP-MEPV. The leaves infiltrated with *A. tumefaciens* containing pSiM24-eGFP-MEPV exhibited substantial phenotypic green fluorescent protein (GFP) expression compared to the leaves infiltrated by *A. tumefaciens* without the plant expression vector (**Figure 1B**).

### 3.2 Extraction and purification of plant-produced MEPV protein

**Figure 2:**
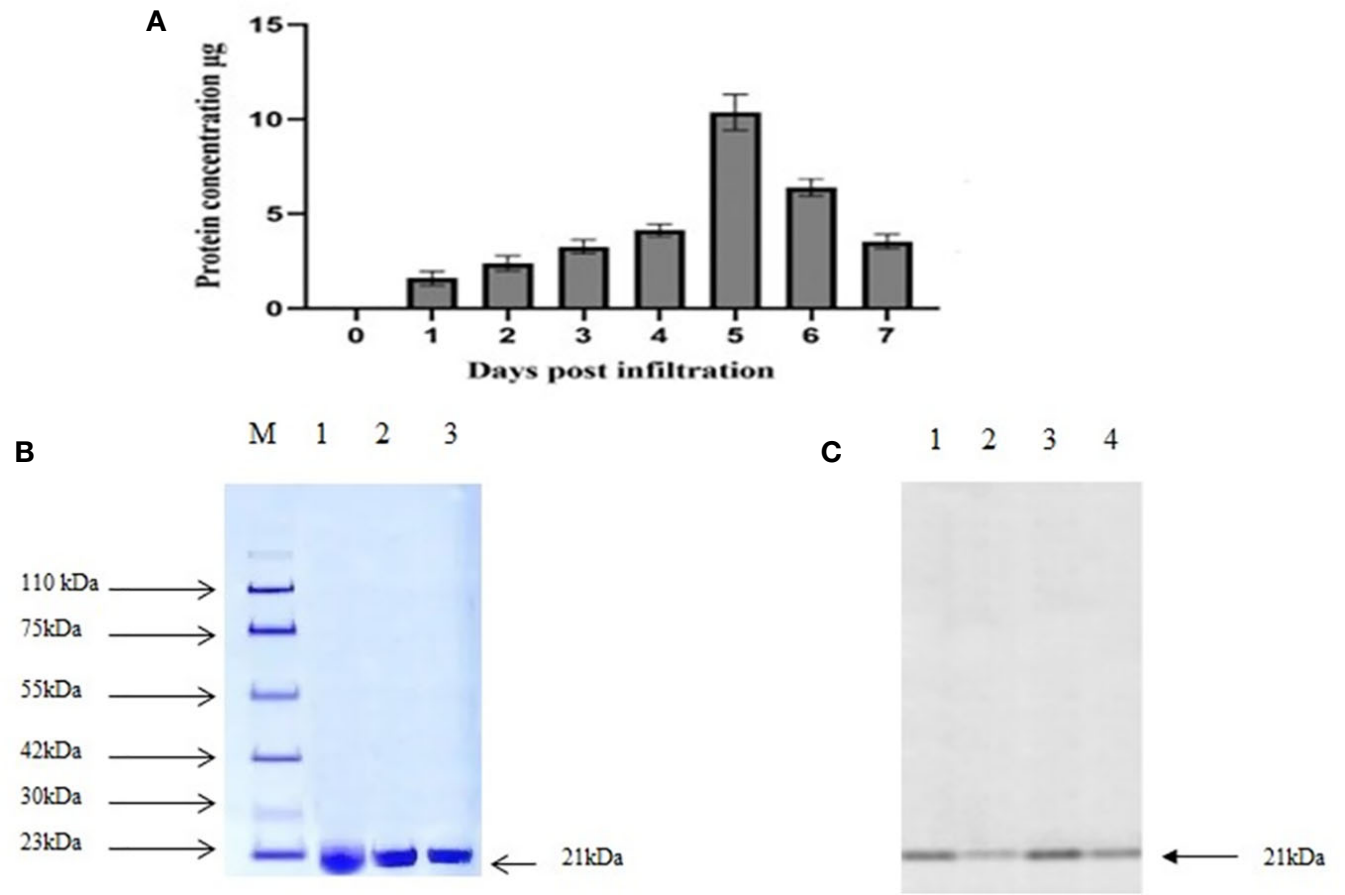
**(A)** Total protein content (µg MEPV/g of fresh weight) concentration. **(B)** SDS analysis of purified protein. M, protein marker; lanes 1 to 3, infiltrated plant leaf sample. **(C)** Western blot analysis of purified protein. Lanes 1 to 4, infiltrated plant leaf sample.

The presence of MEPV protein in agroinfiltrated plant leaves was analyzed using 10% SDS-PAGE. The bands were visible at 21.4 kDa, the expected size of MEPV protein. The total protein content was quantified over a period of 7 days (**Figure 2A**); the highest concentration was observed on day 5.

Plant-produced MEPV protein was extracted and purified by nickel column chromatography, by N-terminal His tag of MEPV protein. The His-tagged fusion proteins were efficiently eluted. In the purified fraction, a prominent single band was observed at 21.4 kDa, which corresponds to the size of MEPV protein (**Figures 2B, C**). The concentration of the purified protein content of MEPV was 10.32 µg/g fresh weight.

### 3.3 Immunogenicity in chicken

**Figure 3:**
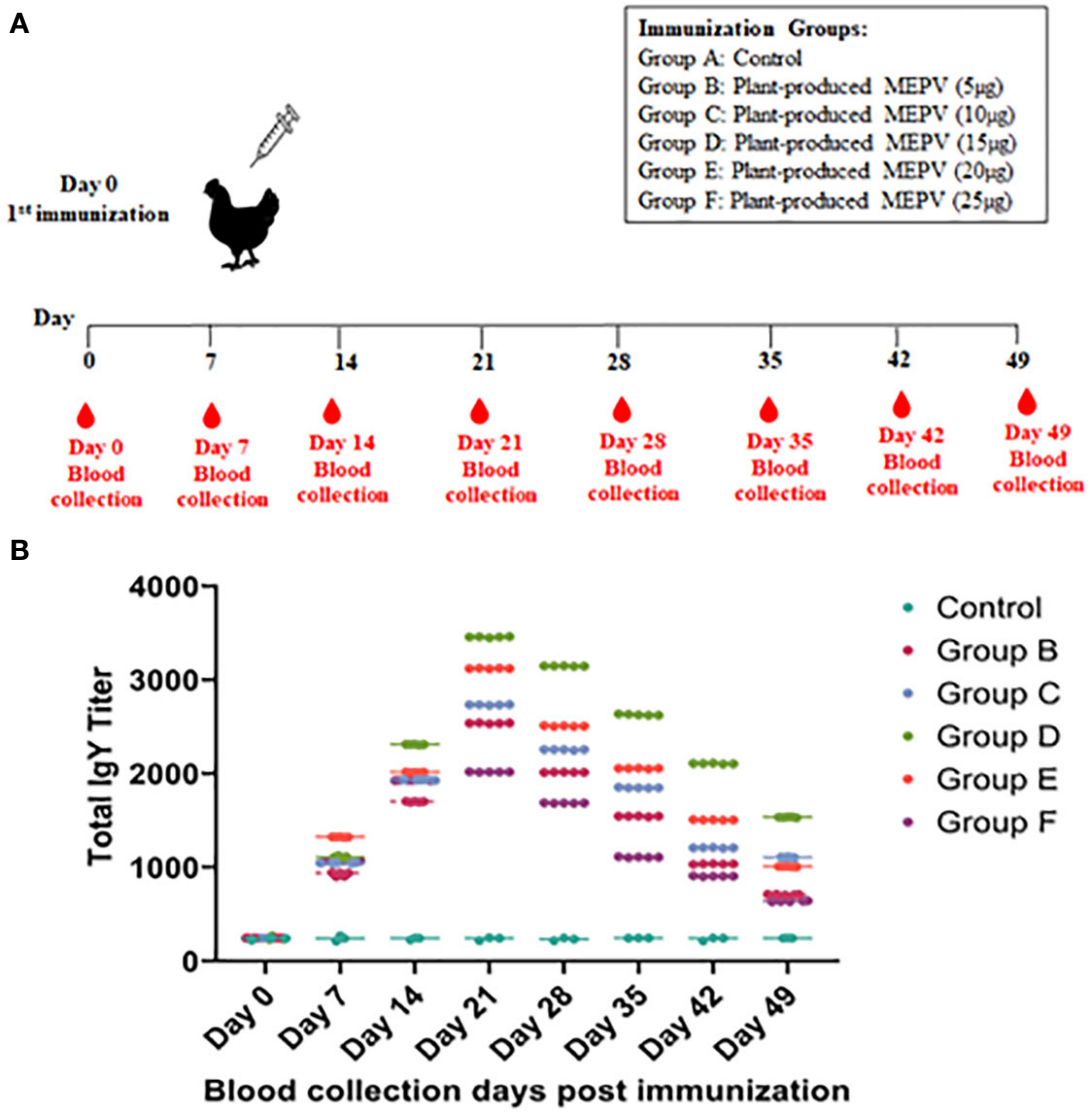
Immunization and blood collection schedule in chickens **(A)**. Chickens were divided into six groups, namely, plant-produced MEPV (5 µg), MEPV (10 µg), MEPV (15 µg), MEPV (20 µg), and MEPV (25 µg) vaccine groups (n = 5) and control group (n = 3). Chickens were immunized on day 0 and were bled on days 0, 7, 14, 21, 28, 35, 42, and 49. The response of MEPV-specific IgY titers in immunized animals is presented **(B)**. Data presented as GMT ± 95% CI of the endpoint titer in each group, n = 5 (control, n = 3). Two-way ANOVA and Tukey’s test were performed.

The titers of total IgY antibodies induced by MEPV in immunized chickens at days 0, 7, 14, 21, 28, 35, 42, and 49 post immunization were measured by ELISA. All chickens immunized with plant-produced MEPV elicited significantly higher antibody titers compared with the control (**Figure 3A**). Higher levels of specific anti-MEPV IgY antibodies were detected in immunized chickens following immunization after day 7, and the highest was observed 21 days after immunization. Likewise, chickens of all six groups (Groups A, B, C, D, E and F) had nearly similar antibody titers on day 0 post immunization without any significant difference. However, after MEPV immunization, the antibody titer in chickens of Group B to Group F was found to be increased progressively as assessed at 7, 14, and 21 days post immunization and started to gradually decrease at 28, 35, 42, and 49 days post immunization. Significant difference (p < 0.0001) in antibody titer was observed between control (Group A) and MEPV-immunized groups (Groups B to F) at all-time points. In Group D (MEPV 15 µg), the serum IgY antibody titers on 14 to 49 days post immunization were comparatively higher when compared with those of other groups (**Figure 3B**). The plant-produced MEPV was able to elicit immune response in chickens; this was validated by the significant increase in the IgY antibody titer in immunized chickens versus control chickens.

## 4 Discussion

To manage the losses caused by chronic respiratory disease attributed to *M. gallisepticum*, the widespread reliance on in-feed antibiotics in the poultry industry has presented challenges. These challenges include the emergence of antibiotic-resistant microbes and the presence of antibiotic residues in meat and eggs (Evans and Hafez, 1992). Current *M. gallisepticum* vaccines are ineffective in eradicating the disease and require proper storage and logistics. Consequently, there is a pressing need to develop a vaccine that is effortless and cost-effective to produce, utilizes scalable technology to meet global demand, and provides immunity against *M. gallisepticum* infection.

Plant molecular farming stands out as an alternative technology for expressing heterologous proteins in plants, facilitating the production of antigens and antibodies. Researchers have explored plants as carriers for vaccine antigen expression, demonstrating their potential for effective and affordable recombinant protein production in large quantities (Mason et al., 1996; Fischer et al., 2004). Plant-expressed vaccine antigens have been shown to trigger a protective immune response (Tacket et al., 2000; Komarova et al., 2010; Jacob et al., 2013). In this study, we utilized *Agrobacterium*-mediated transient expression of an *M. gallisepticum* cytoadherence-based MEPV using the pSiM24-eGFP binary vector.

The first ever plant-produced biopharmaceutical licensed by a governmental agency, the Newcastle disease virus (NDV) poultry vaccine by Dow AgroSciences, was developed in tobacco cell suspension cultures [United States Department of Agriculture (USDA)] (Vermij and Waltz, 2006). Although not released to the public, it marked a pivotal moment for plant-based vaccine approval. Numerous studies have reported the expression of immunogenic hemagglutinin-neuraminidase (HN) protein of NDV in plants, eliciting specific immune responses in chickens and mice (Hahn et al., 2007; Gómez et al., 2009; Ma et al., 2020; Shahid et al., 2020; Nurzijah et al., 2022). Similarly, vaccines for infectious bursal disease virus (IBDV) and the poultry parasite *Eimeria* have been expressed in plants, demonstrating the potential for plant-based vaccine production (Richetta et al., 2017; Lucero et al., 2019; Rage et al., 2020).

Research on plant-based vaccine manufacturing for veterinary diseases has gained interest over the past two decades, offering exciting possibilities such as the development of edible vaccines. This could lead to the creation of prophylactics, vaccinations, and medications that are safe, effective, stable, and affordable for various illnesses, including infectious diseases. Edible vaccines can be produced in large quantities at minimal cost, without the need for a cold chain during transit and storage.

Given the global concern over antibiotic resistance, finding an alternative and efficient control for *M. gallisepticum* infection and its associated losses is crucial. Vaccines based on adhesion and phase variation proteins are considered among the most effective options to prevent and control *M. gallisepticum* infection and maintain poultry health, welfare, and production. However, the success or failure of these vaccines depends on factors such as their type, safety, effectiveness, functional mechanisms, and immunogenicity (Mugunthan et al., 2023).

Comparing multi-epitope-based vaccines to traditional immunizations reveals several advantages. They have a shorter development time, do not require microbial cultivation, and can replace multiple wet lab processes. Unlike live attenuated strains, epitope-based vaccines reduce the risk of virulence reversal. The tiny size of epitopes allows for intelligent alteration and optimization to maximize their efficiency and chemical stability. Their high specialization and stability, coupled with not involving full viruses, make them safe. Multi-epitope vaccines can bind numerous HLA alleles simultaneously, ensuring an appropriate immunological response in a diverse population.

The multi-epitope complementary DNA (cDNA) was successfully cloned into the pSiM24-eGFP plant binary vector, confirmed by PCR and restriction digestion, and transformed into *Agrobacterium*. *Agrobacterium* harboring the MEPV gene was transformed into plant leaves for transient expression through agroinfiltration. The expression of the MEPV gene and proteins in agroinfiltrated leaves was confirmed through PCR and SDS-PAGE, respectively. The plant-expressed MEPV protein was purified by nickel His-tag purification, and its size (21.4 kDa) was validated by Western blot analysis. Immunizing chickens with various concentrations of plant-produced MEPV showed a significant increase in antibody titer in Group D (15 µg MEPV) compared to other groups. Higher levels of specific anti-MEPV IgY antibodies were detected in immunized chickens, with the peak observed on day 21 after immunization, consistently across all six groups (Groups A, B, C, D, E, and F). These results suggest that the anti-MEPV IgY antibodies generated could be used to protect chickens against the disease.

The immunogenic property of plant-expressed MEPV was confirmed by ELISA. This study demonstrates that plants can synthesize *M. gallisepticum*-based MEPV antigens. The use of multi-epitopic antigens appears extremely promising in developing vaccine candidates that generate a lasting protective immune response.

## 5 Conclusion

In conclusion, our study demonstrated that it was feasible to produce MEPV protein in *N. benthamiana* plants using a transient expression system. Furthermore, plant-produced recombinant MEPV protein was shown to be immunogenic in chickens. The vaccine elicited immune responses, suggesting the potential of the plant-produced MEPV as an effective vaccine candidate against CRD caused by *M. gallisepticum*. Collectively, this proof-of-concept study demonstrated that the plant-produced MEPV protein could possibly be further developed as a candidate vaccine.

## Data availability statement

The original contributions presented in the study are included in the article/**Supplementary Material**. Further inquiries can be directed to the corresponding author.

## Ethics statement

The animal study was approved by Thiruvalluvar University Ethical Committe. The study was conducted in accordance with the local legislation and institutional requirements.

## Author contributions

SPM: Conceptualization, Data curation, Formal analysis, Investigation, Methodology, Writing – original draft. DV: Formal analysis, Investigation, Methodology, Data curation, Writing – review & editing. CG: Writing – review & editing, Formal analysis, Validation. DS: Formal analysis, Validation, Writing – review & editing. HMC: Investigation, Supervision, Writing – review & editing.
