# Supplementary figures and images for: A preliminary study of the immunogenic response of plant-derived multi-epitopic peptide vaccine candidate of Mycoplasma gallisepticum in chickens
Source: Front Plant Sci. 2024 Jan 23;14:1298880. doi: 10.3389/fpls.2023.1298880 (PMC10846684; doi:10.3389/fpls.2023.1298880)

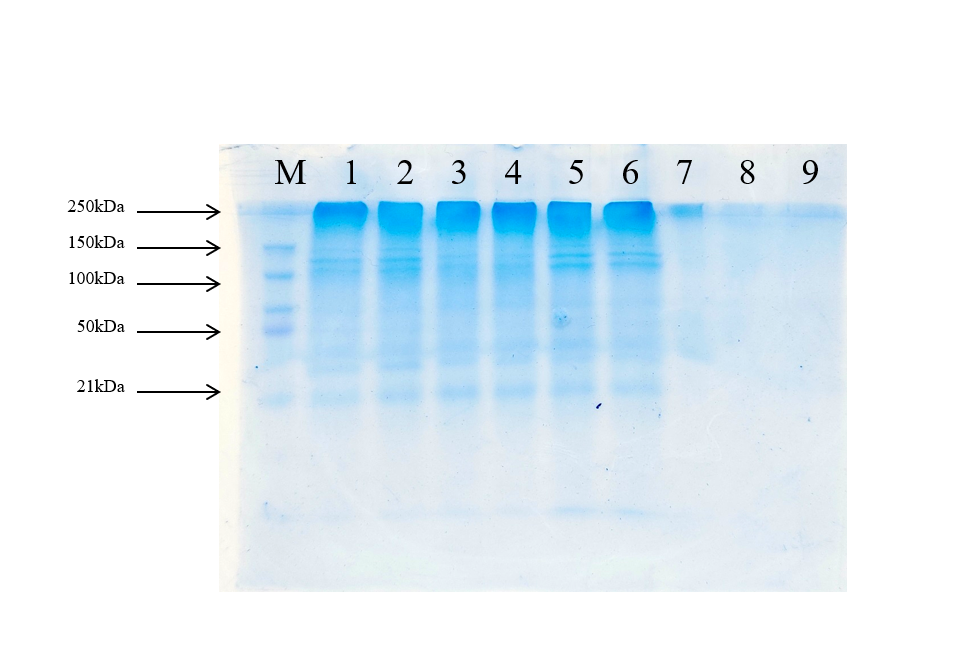

Supplement: Supplementary file 1 [file Image_1.tif]
